# *Salmonella* Pathogenicity Island 1 (SPI-1) and Its Complex Regulatory Network

**DOI:** 10.3389/fcimb.2019.00270

**Published:** 2019-07-31

**Authors:** Lixin Lou, Peng Zhang, Rongli Piao, Yang Wang

**Affiliations:** ^1^Department of Infectious Diseases, First Hospital of Jilin University, Changchun, China; ^2^Department of Pediatrics, University of Oklahoma Health Sciences Center, Oklahoma City, OK, United States; ^3^Department of Gastroenterology, First Hospital of Jilin University, Changchun, China

**Keywords:** *Salmonella*, SPI-1, T3SS, effector, regulation, immune response

## Abstract

*Salmonella* species can infect a diverse range of birds, reptiles, and mammals, including humans. The type III protein secretion system (T3SS) encoded by *Salmonella* pathogenicity island 1 (SPI-1) delivers effector proteins required for intestinal invasion and the production of enteritis. The T3SS is regarded as the most important virulence factor of *Salmonella*. SPI-1 encodes transcription factors that regulate the expression of some virulence factors of *Salmonella*, while other transcription factors encoded outside SPI-1 participate in the expression of SPI-1-encoded genes. SPI-1 genes are responsible for the invasion of host cells, regulation of the host immune response, e.g., the host inflammatory response, immune cell recruitment and apoptosis, and biofilm formation. The regulatory network of SPI-1 is very complex and crucial. Here, we review the function, effectors, and regulation of SPI-1 genes and their contribution to the pathogenicity of *Salmonella*.

## Introduction

The gram-negative bacterial genus *Salmonella* contains as many as six subspecies and more than 2,600 serovars, including numerous serovars pathogenic to humans and a variety of animals (LeLièvre et al., 2019). Salmonellosis, the most frequent foodborne disease in humans, usually results from contaminated water and food. Typhoid fever, caused by *Salmonella enterica* serovar Typhi infection, is still a major health problem, especially in the developing world with substandard water supplies and poor sanitation (Parry et al., 2002; Wain et al., 2015). Better characterization of *Salmonella* has become a hotspot issue. Pathogenic *Salmonella* species invade non-phagocytic intestinal epithelial cells by delivering a specialized set of effectors through sophisticated machinery comprising the type 3 secretion system (T3SS), which plays a crucial role in the pathogenesis of *Salmonella* (Que et al., 2013). *Salmonella* employs two T3SSs encoded by *Salmonella* pathogenicity island 1 (SPI-1) and *Salmonella* pathogenicity island 2 (SPI-2). SPI-1 is a gene cluster and consists of a 40-kb region, which includes 39 genes encoding T3SS-1 and its chaperones and effector proteins as well as some transcriptional regulators that control the expression of many virulence genes located within and outside SPI-1 (Hansen-Wester and Hensel, 2001; Zhang K. et al., 2018). T3SS-1 of *Salmonella* can affect the phenotype, polarization and function of macrophages (Kyrova et al., 2012; Zhao et al., 2018). The ubiquity of SPI-1 is conserved and required for *Salmonella* virulence, demonstrated by its active role in the entry process. Further studies have revealed that the SPI-1-encoded T3SS has additional functions and that its regulatory network is very complex. This review focuses on the effect and the regulation of SPI-1 and the relationship between host immunology and SPI-1 in *Salmonella*.

## The Role of SPI-1

**Figure 1:**
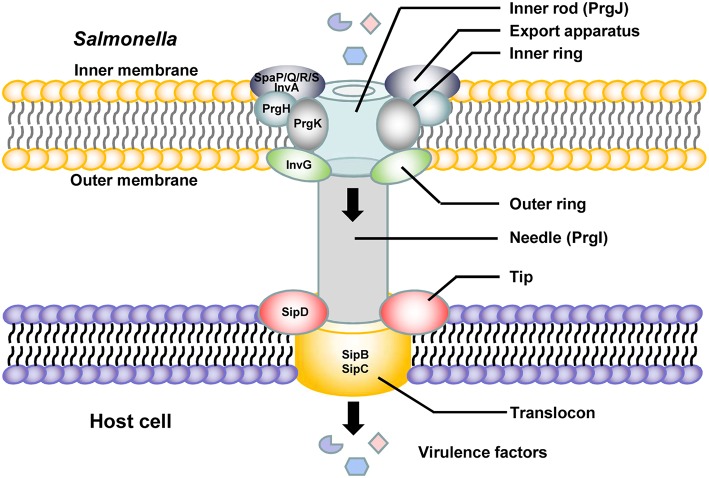
Schematic diagram of the SPI-1-related T3SS needle apparatus in contact with a host cell.

*Salmonella* pathogenicity island 1 (SPI-1) plays a crucial role in the interaction between *Salmonella* and host cells. SPI-1 promotes *Salmonella* invasion into epithelial cells (Raffatellu et al., 2005). The T3SS is assembled from the proteins encoded by SPI-1 and is termed the needle complex. Translocases and effector proteins are delivered into host cells through the needle complex. The needle complex spans the bacterial envelope, and a needle-like extension protrudes from the bacterial inner and outer membranes to the host cell membranes (Kubori et al., 1998; Sukhan et al., 2001). There are several highly conserved proteins and an ATPase in the needle complex, and all of them are essential for secretion (Figure 1). A sorting platform determines the order of protein secretion in the SPI-1 T3SS of *Salmonella*. The sorting platform consists of five proteins, SpaO, OrgA, OrgB, InvI, and the hexameric ATPase InvC, in *Salmonella*. Type III secretion chaperones are required for loading effectors and translocases onto the sorting platform (Lara-Tejero et al., 2011). The needle complex is composed of a multiple-ring cylindrical base. The needle complex base is initiated at the export apparatus, which is composed of the proteins InvA, SpaP, SpaQ, SpaR, and SpaS (Cornelis, 2006; Galán and Wolf-Watz, 2006; Minamino et al., 2008; Worrall et al., 2010). The export apparatus is essential to the assembly and/or the stability of the needle complex base (Wagner et al., 2010). Three proteins, InvG, which comprises the outer rings; PrgH, and PrgK, which are thought to form the rest of the structure, constitute the base with equimolar amounts. PrgI is the main component of the needle portion (Kubori et al., 2000; Marlovits et al., 2004; Schraidt et al., 2010). The length of the needle segment is controlled by the protein InvJ (Kubori et al., 2000). PrgJ forms an inner rod within the basal body and the needle is anchored by that inner rod, which forms a conduit between the bacterial cytoplasm and the host cell membrane (Galán and Wolf-Watz, 2006). The needle tip structure is capped with SipD, which is secreted by a nascent T3SS filament. The tip protein SipD is stably bound at the tip of the needle formed by a polymer of the protein PrgI. The needle tip complex regulates the secretion of effectors from *Salmonella* into the host cell (Lunelli et al., 2011). Upon host cell contact, the protein SipD forms a platform for the translocon composed of the transmembrane proteins SipB and SipC and interacts with their N-terminal ectodomains (Lara-Tejero and Galán, 2009; Kaur et al., 2016; Glasgow et al., 2017). SipB is a *Salmonella* translocon protein that is inserted into host membranes to form a channel associated with SipD at the needle tip, through which T3SS effectors are translocated into the host cell (Myeni et al., 2013; McShan et al., 2016). These translocons, encoded by *Salmonella* SPI-1, play an important role in both *Salmonella* contact with and invasion of host cells and the colonization of mammalian intestinal epithelial cells (Knodler and Steele-Mortimer, 2003; Boyen et al., 2006; Sivula et al., 2008; Lara-Tejero and Galán, 2009).

The SPI-1-encoded proteins are also required for the complex immune responses of host cells during *Salmonella* infection. *Salmonella* SPI-1 induces neutrophil recruitment during enteric colitis, leading to a reduction and alteration in intestinal microbiota (Sekirov et al., 2010). The SPI-1-encoded T3SS is required not only for cell invasion but also for suppression of early proinflammatory cytokine expression in macrophages, including that of IL-1β, IL-8, TNF-α, IL-23α, GM-CSF, and IL-18 (Pavlova et al., 2011). SPI-1 is involved in MHC-II downregulation and polarization to the M2 phenotype in macrophages (Kyrova et al., 2012; Van Parys et al., 2012; Zhao et al., 2018). *Salmonella* can cross the blood-brain barrier and reach various brain tissues because the SPI-1 and outer membrane protein A genes of *Salmonella* increase penetration of the blood-brain barrier (Chaudhuri et al., 2018).

## The Effector Proteins of the SPI-1 T3SS

Many gram-negative bacterial pathogens use a T3SS to inject their own proteins, termed effectors, into host cells to modulate some cellular functions (Hueck, 1998). Many SPI-1 effector proteins have been identified in *Salmonella*. These effectors play a variety of roles during *Salmonella* infection, including taking part in rearrangement of the host cytoskeleton, immune cell recruitment, cell metabolism, fluid secretion, and regulation of the host inflammatory response (Collier-Hyams et al., 2002; Brawn et al., 2007; Myeni et al., 2013). Several SPI-1 translocated effectors are responsible for the invasion of epithelial cells (Fu and Galán, 1999; Hayward and Koronakis, 1999; Mirold et al., 2001a,b). *Salmonella* expresses different SPI-1 effectors when colonizing specific tissues. The level and timing of the expression of these proteins determine the consequences of *Salmonella* infection and might be essential for tissue-specific aspects of its pathogenesis (Gong et al., 2009, 2010). The differential stability of some effector proteins (SopE and SptP) is central to the regulation of the activity of bacterial effectors within host cells (Kubori and Galán, 2003). We describe some of the effector proteins of SPI-1 T3SS and their functions below.

(1) AvrA

The virulence-associated gene *avrA* is located within SPI-1 and exists in most *Salmonella* strains (Amavisit et al., 2003). AvrA is a multifunctional enzyme and plays a critical role in inhibiting activation of the key proinflammatory NF-κB transcription factor and apoptosis via the JNK pathway (Collier-Hyams et al., 2002; Jones et al., 2008; Liao et al., 2008; Wu et al., 2012). It is phosphorylated in mammalian cells, and its phosphorylation requires the extracellular-regulated kinase (ERK) signaling pathway (Du and Galán, 2009). AvrA promotes intestinal epithelial cell proliferation (Ye et al., 2007) and tumorigenesis (Lu et al., 2010) by blocking the degradation of IκBα and β-catenin. It enhances the development of infection-associated colon cancer by activating the STAT3 signaling pathway (Lu et al., 2016). AvrA expression in *Salmonella* stabilizes the structure and influences the function of tight junctions of intestinal epithelial cells via the JNK pathway, while its expression increases bacterial invasion ability and translocation (Liao et al., 2008; Lu et al., 2010; Lin et al., 2016).

(2) Sips

There are four *Salmonella* invasion proteins (Sips), namely, Sips A–D. These Sips are exported and translocated into the host cell plasma membrane or cytosol and play essential and complex roles in the secretion and translocation of SPI-1 effectors. SipA is an actin-binding protein and enhances the efficiency of the entry process of *Salmonella* into host cells by influencing different stages in the formation of membrane ruffles and rearrangement of the actin cytoskeleton (Zhou et al., 1999). SipA regulates the concentration, polymerization and stability of the actin molecules at the site of bacterial entry and increases the bundling activity of host cell fimbrin (Galan and Zhou, 2000; McGhie et al., 2001, 2004). SipA is not essential for uptake, but it enhances the efficiency of the entry process (Zhou et al., 1999). SipA is exposed on the cytoplasmic face of the *Salmonella*-containing vacuole (SCV) after *Salmonella* internalization in both non-phagocytic cells and macrophages, and it is involved in the regulation of phagosome maturation and intracellular *Salmonella* replication (Brawn et al., 2007). The N-terminal domain of SipA induces polymorphonuclear leukocyte recruitment (Lee et al., 2000; Wall et al., 2007). SipA causes the activation and release of caspase-3, which plays multiple roles in the immune response of host cells, including in apoptosis, differentiation, proliferation, immunomodulation, immune cell migration, and signal transduction (Srikanth et al., 2010; McIntosh et al., 2017). SipB, SipC, and SipD are translocon proteins that participate in the formation of the SPI-1 T3SS needle complex (Figure 1; Zierler and Galán, 1995; Collazo and Galán, 1997; Scherer et al., 2000; Myeni and Zhou, 2010; Myeni et al., 2013). SipB is necessary for *Salmonella*-induced caspase-1-dependent apoptosis and the release of IL-18 (Hersh et al., 1999; Dreher et al., 2002; Obregon et al., 2003). SipC is a *Salmonella* translocon protein that targets F-actin, which is necessary for pathogen internalization (Kaniga et al., 1995) and promotes *Salmonella* invasion. Antibodies against SipD inhibit *Salmonella* invasion, and SipD might be a potential target for blocking SPI-1-mediated virulence (Desin et al., 2010). The N-terminal domain of SipD promotes the secretion of effectors and functions at the post-transcriptional and post-translational levels (Glasgow et al., 2017).

(3) SptP

*Salmonella* protein tyrosine phosphatase (SptP) was identified in 1996. The translocation of SptP to host cells results in the disruption of the cellular actin cytoskeleton (Kaniga et al., 1996; Fu and Galán, 1998a). However, SptP is directly responsible for the reversal of the actin cytoskeletal changes induced by other effectors of *Salmonella* via regulating villin phosphorylation (Fu and Galán, 1999; Lhocine et al., 2015; Johnson et al., 2017). SptP translocation occurs during entry, when it downregulates membrane ruffling and then downmodulates ERK and mitogen-activated protein kinase (MAPK) activation and the secretion of proinflammatory cytokines induced by *Salmonella* entry (Kubori and Galán, 2003; Lin et al., 2003; Eswarappa et al., 2008; Button and Galán, 2011; Johnson et al., 2017). Protein SicP, which is immediately upstream of SptP, acts as a chaperone for SptP. Coupling of their translation is required for maximally efficient secretion of SptP (Fu and Galán, 1998b; Zhou and Galán, 2001; Button and Galán, 2011). SptP-mediated dephosphorylation of valosin-containing protein promotes *Salmonella* intracellular replication (Humphreys et al., 2009). SptP suppresses the degranulation and activation of mast cells, which enables bacterial dissemination. It is a powerful mechanism utilized by *Salmonella* to impede early innate immunity (Choi et al., 2013; Kawakami and Ando, 2013).

(4) Sops

The *Salmonella* outer proteins (Sops) are effector proteins that consist of SopA, SopB, SopD, SopD2, SopE, and SopE2. Sops are involved in the control of different stages of polymorphonuclear leukocyte influx and rearrangement of the cytoskeleton (Wood et al., 1996, 2000; Galyov et al., 1997; Jones et al., 1998; Bakshi et al., 2000; Boyle et al., 2006; Schlumberger and Hardt, 2006), contribute to *Salmonella* invasion and are responsible for inducing inflammation and diarrhea (Wood et al., 2000; Raffatellu et al., 2005; Zhang et al., 2005). *sopA, sopB*, and *sopE* are regulated cooperatively by HilA and InvF (Thijs et al., 2007).

SopA can induce fluid secretion and the inflammatory response in *Salmonella*-infected intestines after being translocated into host cells (Wood et al., 2000). The stability and translocation of SopA requires the chaperone InvB (Ehrbar et al., 2004). Efficient bacterial escape from the SCV to the cytosol of epithelial cells requires HsRMA1-mediated SopA ubiquitination and contributes to *Salmonella*-induced enteropathogenicity. HsRMA1 is a membrane-bound ubiquitin E3 ligase, although SopA is an E3 ligase itself (Zhang et al., 2005, 2006). SopA regulates innate immune responses by mediating the ubiquitination and proteasomal degradation of tripartite-motif containing (TRIM) E3 ligases (TRIM56 and TRIM65) (Kamanova et al., 2016; Fiskin et al., 2017).

SopB/SigD, an inositol phosphatase, is required for fluid and chloride secretion and neutrophil recruitment (Norris et al., 1998; Bertelsen et al., 2004). It mediates virulence by interdicting inositol phosphate signaling pathways and inducing Akt activation (Norris et al., 1998; Steele-Mortimer et al., 2000; Marcus et al., 2001). SopB/SigD also has antiapoptotic activity and is related to intracellular replication because of the sustainment of Akt activation (Knodler et al., 2005; Rodríguez-Escudero et al., 2011; García-Gil et al., 2018). SopB/SigD promotes membrane fission and damage to epithelial barrier function during invasion (Marcus et al., 2002; Terebiznik et al., 2002; Bertelsen et al., 2004). It stimulates nitric oxide (NO) production (Drecktrah et al., 2005).

SopD affects multiple signals and protein interactions and contributes to the systemic virulence of *Salmonella* and the development of gastroenteritis (Galyov et al., 1997; Jones et al., 1998; Galan and Zhou, 2000; Boonyom et al., 2010). It is involved in membrane fission and macropinosome formation during *Salmonella* invasion, with cooperation from SopB (Bakowski et al., 2007). SopD and SopD2 promote bacterial replication in host cells and are related to the SCV (Jiang et al., 2004; Bakowski et al., 2007; Maserati et al., 2017). SopD2 contributes to *Salmonella*-induced filament formation (Jiang et al., 2004) and inhibits the vesicular transport and tubule formation that extend outward from the SCV (Schroeder et al., 2010).

SopE, a Rho GTPase exchange factor, induces rapid actin cytoskeleton rearrangements, membrane ruffling, and consequent pathogen macropinocytosis and promotes bacterial invasion (Wood et al., 1996; Hardt et al., 1998; Rudolph et al., 1999; Galán and Fu, 2000; Mirold et al., 2001b; Humphreys et al., 2012; Lim et al., 2014). SopE transiently localizes to the early SCV and contributes to intracellular replication (Vonaesch et al., 2014). SopE2, which is homologous to SopE, has similar mechanisms of action to those of SopE (Bakshi et al., 2000; Stender et al., 2000; Friebel et al., 2001; Mirold et al., 2001a; Schlumberger and Hardt, 2006). SopE is rapidly degraded by a proteasome-mediated pathway, whereas SptP is slowly degraded, which inactivates Cdc42 and Rac1 and thereby reverses SopB-, SopE-, and SopE2-signaling (Fu and Galán, 1999; Kubori and Galán, 2003; Van Engelenburg and Palmer, 2008; Vonaesch et al., 2014). SopE induces the host to produce nitric oxide synthetase (iNOS) in the intestine, leading to intestinal inflammation (Bliska and van der Velden, 2012).

## The Regulation of SPI-1

**Figure 2:**
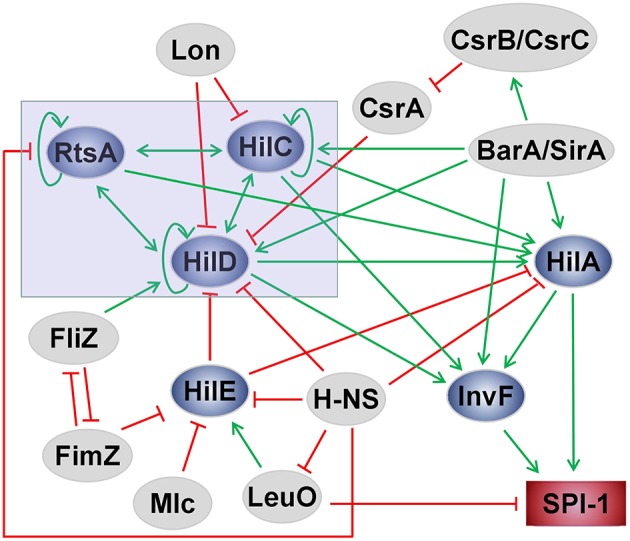
Scheme of the SPI-1 regulatory network in *Salmonella*. The green arrows indicate activation, and the red lines with flat ends represent inhibition.

*Salmonella* pathogenicity island 1 (SPI-1) plays a crucial role not only in the colonization and invasion of *Salmonella* in the gut but also in the induction of neutrophil recruitment (Boyen et al., 2006). The regulation of the process involves many environmental stimuli and genetic regulators in complex networks (Figure 2). Several transcriptional regulators (e.g., HilA, HilC, HilD, and InvF) are encoded by SPI-1. Induction of SPI-1 requires the expression of *invF* and *hilA* because they are transcriptional activators of SPI-1 genes (Altier, 2005; Jones, 2005; Ellermeier and Slauch, 2007). The feed-forward regulatory loop of HilC–RtsA–HilD is the most important core part of the regulatory networks to control the transcription of *hilA*, while HilA is the central regulator of SPI-1 (Ellermeier et al., 2005; Dieye et al., 2007). HilA directly activates the expression of two SPI-1 genes (*invF* and *sicA*) that encode SPI-1 T3SS apparatus components. InvF, a transcriptional activator of the AraC family, activates the expression of SPI-1 T3SS effectors encoded both inside and outside of SPI-1 (Darwin and Miller, 1999; Eichelberg and Galán, 1999). The activity of InvF requires SicA, which is also encoded within SPI-1 (Darwin and Miller, 2000, 2001). Each activator among HilC, RtsA, and HilD can bind to the *hilA* promoter to activate the expression of *hilA*, and HilA can also induce its own expression significantly as well as activate the other two regulators (Schechter and Lee, 2001; Boddicker et al., 2003; Ellermeier et al., 2005). Furthermore, they can activate the expression of *invF* in a HilA-independent manner (Akbar et al., 2003; Baxter et al., 2003). HilE is the most important negative regulator of *hilA* expression. HilE represses the SPI-1 genes by binding to HilD, thus inactivating HilD and preventing the activation of HilA (Paredes-Amaya et al., 2018). Many other regulators can influence SPI-1 through interacting with the core network. Mlc, a global regulator of carbohydrate metabolism, controls several genes related to sugar utilization. Mlc downregulates *hilE* expression by binding to the *hilE* P3 promoter (Lim et al., 2007). SirA, a member of the phosphorylated response regulator protein family, positively regulates the HilD–HilC–RtsA–HilA network by activating HilA, HilC, or HilD (Behlau and Miller, 1993; Johnston et al., 1996; Teplitski et al., 2003; Ellermeier and Slauch, 2007). The action of BarA is coupled to SirA. In many studies, BarA/SirA is regarded as a two-component regulator that activates *hilA* expression and can also activate the *invF* gene without HilA involvement (Johnston et al., 1996; Rakeman et al., 1999; Altier et al., 2000; Teplitski et al., 2003). CsrA, a global regulatory RNA-binding protein, post-transcriptionally downregulates *hilD* expression by binding near the translation initiation codon sequences of the *hilD* mRNA directly, preventing HilD translation and leading to *hilD* mRNA turnover (Lucchetti-Miganeh et al., 2008; Martínez et al., 2011). The negative regulation is counteracted by the BarA/SirA two-component system, which directly activates the expression of *csrB/C*, two non-coding regulatory RNAs that sequester CsrA, thereby preventing CsrA from binding to its target mRNAs (Teplitski et al., 2003; Timmermans and Van Melderen, 2010; Martínez et al., 2011, 2014; Potts et al., 2019). H-NS is an abundant DNA-binding protein found in enteric bacteria, including *Salmonella* (Marsh and Hillyard, 1990; Owen-Hughes et al., 1992). H-NS inhibits the core positive regulators of SPI-1, including HilA, HilD, and RtsA, thus inhibiting the expression of SPI-1 as well as that of many other A + T-rich genes or ancestral DNA (Van Velkinburgh and Gunn, 1999; Lucchini et al., 2006; Navarre et al., 2006). The repression effect on *rtsA* is the most efficient among them. HilD, HilC, and RtsA bind to a common site in the rtsA promoter and antagonize H-NS-mediated repression (Schechter et al., 2003; Olekhnovich and Kadner, 2007). Interestingly, H-NS also represses the promoters of *leuO* and *hilE*, which are regarded as negative regulatory genes. HilE downregulates the expression of SPI-1 by directly inactivating HilD (Baxter et al., 2003). LeuO, a LysR-type transcriptional regulator, has been identified as a *Salmonella* virulence factor through genetic screening (Tenor et al., 2004; Lawley et al., 2006). The regulatory effect of LeuO is concentration-dependent (Dillon et al., 2012; Hernández-Lucas and Calva, 2012). LeuO is regarded as a transcriptional antagonist of H-NS because some genes repressed by H-NS can be activated by LeuO (Hernández-Lucas et al., 2008; Shimada et al., 2009). LeuO inhibits the expression of SPI-1 mainly by directly activating the promoter of *hilE* and via an unknown HilE-independent mechanism (Espinosa and Casadesús, 2014). However, LeuO has also been suggested to play a backup role for H-NS. The inhibitory effect of LeuO on SPI-1 genes may occur under growth conditions where H-NS does not perform such activity (Fahlen et al., 2000). FliZ, a flagellar regulator, can inhibit the expression of the type-1 fimbrial gene through post-transcriptional regulation of FimZ. FimZ is a regulator known to facilitate fimbrial protein expression and to repress the expression of flagellar genes (Saini et al., 2010). FliZ post-transcriptionally controls HilD to upregulate *hilA* expression (Chubiz et al., 2010). FimZ enhances the expression of *hilE*, which negatively regulates *hilD*. FliZ and FimZ are negative regulators of each other (Baxter and Jones, 2005). *glnA*, the glutamine synthetase gene, is essential for the growth and virulence of *Salmonella* because it upregulates FliZ, HilA, and HilD levels, improving the expression of SPI-1-associated effector genes, such as *sopA, sopB, sopD*, and *invF* (Aurass et al., 2018). The global regulatory system ArcAB promotes the expression of genes associated with the SPI-1 T3SS, such as *invF, hilA*, and *sipC*. It participates in *Salmonella* adaptation to changing oxygen levels. ArcAB is also involved in promoting bacterial intracellular survival (Lim et al., 2013; Pardo-Esté et al., 2018).

Because environmental changes, such as osmolarity, pH, and oxygen tension, influence the expression of *hilA* and because constitutive expression of *hilA* substantially frees invasion genes from the control of these environmental signals, it has been supposed that HilA plays a central role in the coordinated environmental regulatory effects of invasion genes (Bajaj et al., 1996). Bile, Mg^2+^ concentration and short-chain fatty acids can also regulate invasion (Altier, 2005). Bile is produced continuously by the liver and is involved in the digestion and absorption of fats. Bile is stored in the gall bladder at high concentrations prior to release into the intestines and serves as an important environmental cue to upregulate virulence gene expression during infection within the host gastrointestinal tract. *Salmonella* controls the production of virulence factors following bile exposure. The bile presents different regulatory effects on the SPI-1 T3SS between non-typhoidal and typhoidal *Salmonella*. The expression and activity of the *S. Typhimurium* SPI-1 T3SS are repressed by bile via BarA/SirA (Prouty and Gunn, 2000; Ellermeier and Slauch, 2007), while those of *S. Typhi* are increased by bile via prolonging the half-life of HilD and increasing SipC, SipD, SopB, and SopE expression (Johnson et al., 2018). Both *phoPQ* and *phoBR*, two-component systems, are very important regulators of *hilA* expression. These environmental signals could influence the expression and phosphorylation of *fimZ*. Under conditions of low Mg^2+^ concentration, the PhoPQ regulon is activated, leading to the phosphorylation of FimZ with a subsequent increase in *hilE* expression. Under conditions of low phosphate, PhoBR is activated, which increases *fimZ* expression, thus upregulating *hilE* expression (Baxter and Jones, 2015). The concentrations and composition of short-chain fatty acids regulate the SPI-1 T3SS via BarA/SirA (Lawhon et al., 2002). Propionate represses the SPI-1 T3SS by reducing the stability of HilD through post-translational modification (Hung et al., 2013). Lon protease, a negative regulator of SPI-1 genes, is important for the downregulation of *hilA* expression and intracellular survival after the invasion of epithelial cells through the degradation of HilC and HilD (Boddicker and Jones, 2004; Takaya et al., 2005). LoiA directly represses *lon* expression to activate the expression of SPI-1 genes (Jiang et al., 2017, 2019; Li et al., 2018). *Salmonella* can sense sugar availability by Mlc. The relatively high glucose concentration in the proximal small intestine can inhibit SPI-1 gene expression via Mlc, perhaps together with PhoBR and/or SirA (Agbor and McCormick, 2011). Lysophosphatidylcholine released following caspase-1 activation in *Salmonella*-infected cells promotes the expression of Sips and HilA and increases *Salmonella* invasion of host cells, and it is regarded as a key component of a novel regulatory mechanism for the regulation of cellular invasion with pathogenic *Salmonella* (Shivcharan et al., 2018).

Some small molecule compounds have been found to have an effect on the regulation of SPI-1. Dimethyl sulfide inhibits the expression of multiple SPI-1-related genes, including *hilA, invA, sopA, sopB*, and *sopE2* (Antunes et al., 2010). L-arabinose, a plant-derived sugar, may serve as an inhibitory signal for SPI-1 of *Salmonella* by inhibiting the expression of *hilD* under certain circumstances (López-Garrido et al., 2015). *Bifidobacterium thermophilum* RBL67, a human fecal isolate, upregulates the expression of SPI-1-related genes of *Salmonella*, including *sipB, sipD, prgI/H/K, invA/C/B/G/H, spaS/R/Q/P/O*, and *sicA/P*. However, it also activates some genes located on SPI-2 and fimbrial genes, leading to redundant energy expenditure and protective activity against *Salmonella* infection (Tanner et al., 2016). Seaweed water extracts (*Sarcodiotheca gaudichaudii* and *Chondrus crispus*) can suppress expression of the SPI-1-associated genes *hilA, sipA*, and *invF* and may also impart beneficial effects on animal and human health (Kulshreshtha et al., 2016). Cytosporone B can decrease the expression of *hilC, hilD, rtsA, hilA, sipA*, and *sipC*. It regulates the transcription of SPI-1-related genes through the Hha–H-NS–HilD–HilC–RtsA–HilA regulatory pathway and has potential benefits in anti-*Salmonella* drug discovery (Li et al., 2013). Sanguinarine chloride, a natural compound, downregulates the transcription of HilA and consequently decreases the production of SipA and SipB. Sanguinarine chloride inhibits the invasion of host cells by *Salmonella*. It is a putative SPI-1 inhibitor and could be a promising anti-*Salmonella* compound (Zhang Y. et al., 2018). Methylthioadenosine reduces the virulence of *Salmonella* by suppressing the expression of *invF* and *sipB* (Bourgeois et al., 2018). Some kinds of prenylated flavonoids show a strong inhibitory effect on the secretion of SPI-1 effector proteins through regulating the transcription of *sicA*/*invF* and the transportation of the effector proteins SipA/B/C/D (Guo et al., 2016). Baicalein, a specific flavonoid from *Scutellaria baicalensis*, targets SPI-1 effectors and translocases to inhibit *Salmonella* invasion. It does not suppress SPI-1-related proteins directly but affects the assembly, stability, or activity of their substrates (Tsou et al., 2016). Quercetin, another naturally occurring flavonoid, can also antagonize SPI-1 T3SS substrates of *Salmonella* (Tsou et al., 2016). Biochanin a, a major isoflavone constituent found in red clover, cabbage, alfalfa, and some other herbal dietary supplements, suppresses the expression of *sipA, sipB, sipC, hilA*, and *hilD* and reverses macrophage polarization via downregulating SPI-1 expression (Zhao et al., 2018). Indole, a microbial metabolite of tryptophan, inhibits *Salmonella* invasion by decreasing SPI-1-related gene expression, including that of *hilA, prgH, invF*, and *sipC*, via both PhoPQ-dependent and -independent mechanisms (Kohli et al., 2018). These compounds and medicines may have immunomodulatory effects on *Salmonella*-infected host cells and regulate their bactericidal activity. They might be promising candidates for novel types of anti-*Salmonella* drugs.

## Conclusion

The virulence-associated SPI-1 has been widely explored in interactions between *Salmonella* and its hosts. SPI-1 affects the whole process of salmonellosis, including pathogen invasion, proliferation, and host responses. Greater insights into SPI-1 and its complex regulatory network might contribute to drug investigation and *Salmonella* infection control.

## Author Contributions

YW conceived the general idea. LL, PZ, and RP conducted the literature study and wrote the draft manuscript. YW provided critical revision and final approval of the manuscript.

### Conflict of Interest Statement

The authors declare that the research was conducted in the absence of any commercial or financial relationships that could be construed as a potential conflict of interest.
